# A novel method to characterize silica bodies in grasses

**DOI:** 10.1186/s13007-016-0108-8

**Published:** 2016-01-21

**Authors:** Clemon Dabney, Jason Ostergaard, Eric Watkins, Changbin Chen

**Affiliations:** Department of Horticultural Science, University of Minnesota, 1970 Folwell Avenue, Alderman Hall, Saint Paul, MN 55108 USA

**Keywords:** Silica body, Fluorescence microscopy, Dry ash-imaging, Photoshop, *Koeleria macrantha*, Junegrass

## Abstract

**Background:**

The deposition of silicon into epidermal cells of grass species is thought to be an important mechanism that plants use as a defense against pests and environmental stresses. There are a number of techniques available to study the size, density and distribution pattern of silica bodies in grass leaves. However, none of those techniques can provide a high-throughput analysis, especially for a great number of samples.

**Results:**

We developed a method utilizing the autofluorescence of silica bodies to investigate their size and distribution, along with the number of carbon inclusions within the silica bodies of perennial grass species *Koeleria macrantha*. Fluorescence images were analyzed by image software Adobe Photoshop CS5 or ImageJ that remarkably facilitated the quantification of silica bodies in the dry ash. We observed three types of silica bodies or silica body related mineral structures. Silica bodies were detected on both abaxial and adaxial epidermis of *K. macrantha* leaves, although their sizes, density, and distribution patterns were different. No auto-fluorescence was detected from carbon inclusions.

**Conclusions:**

The combination of fluorescence microscopy and image processing software displayed efficient utilization in the identification and quantification of silica bodies in *K. macrantha* leaf tissues, which should applicable to biological, ecological and geological studies of grasses including forage, turf grasses and cereal crops.

## Background

The epidermal cells of grasses (Poaceae) are arranged in parallel rows with combinations of diverse cell types [1]. Some of these cells are specific for silicon (Si) deposition and are called silica cells. The Silicon accumulated in the silica cells develops into the mineral structures of amorphous hydrated silica (SiO_2_·nH_2_O) having various shapes and properties called silica bodies, silica phytoliths, or plant opal [2–4].

Silica bodies are one of the most durable structures in grass tissues that remain as particles in the soil even after all other organic parts of plant have naturally decayed or degraded. These particles in the soil and ash can be very important research tools for systematic botanists [2, 5], environmental biologists [6], archeologists [4, 7, 8], paleontologists/paleobotanists [9–14], and geologists [15–17].

The amounts of silica in plant tissues suggest that silicon has a very important role in growth and development. For example, in rice (*Oryza sativa* L.), several fold more Si can be detected in shoots compared with the amounts of nitrogen, phosphorus, or potassium [18], reaching up to ten percent of its dry mass [19, 20].

Functional analyses of plant silica have shown that silicon is critical for mitigating stressors such as fungal infection [21, 22], herbivory [23, 24], wear [25, 26], and drought [27–30]. Mature silica bodies have been found to deter herbivory and increase the abrasiveness of grass leaf blades [31–33]. In addition, ample silica bodies have been associated with photosynthetic activities [29, 34, 35], although the mechanism for this response remains unclear [35].

Because we are interested in improving stress tolerance response in turf grasses, we wanted to develop a method to efficiently identify and quantify silica bodies in perennial grasses. Such a method could also be extended to other grass species, such as important forage grasses and cereals. In searching for an easy, economical, and fast method to study the morphology and distributional patterns of silica bodies in turf grasses and other plants, we found a number of available techniques. These include dry ash method, wet oxidation method, scanning electron microscopy (SEM) method, and X-ray image analysis. Among which, dry ash-imaging is one of the most commonly used methods for studying silica bodies in modern plants. To study grass leaves, ash imaging has been a method-of-choice to many researchers; however, this method is extremely labor intensive when analyzing the size, density, and distribution patterns using brightfield light microscopy and researchers have to manually measure a great number of silica bodies in order to perform a statistically meaningful analysis [2, 36]. This method can be accomplished by placing samples in porcelain crucibles and into a muffle furnace, or an oven, for 1–2 h at 500 °C, but some morphological changes might occur to certain, lightly silicified phytoliths when the temperature exceeds 600 °C [2, 4, 36, 37]. The wet oxidation method was developed to examine the isolated silica bodies and is suitable for measuring the abundance of silica bodies in plant tissues, but does not work well for analyzing the distribution patterns of silica bodies [2, 4, 38]. In comparison to the dry ash method, the wet oxidation method results in less damaged silica bodies, especially when the samples are exposed in an environment of 600 °C or higher [39]. Due to the limitation of applying light microscopy to examine surface morphology at extra high magnification, scanning electron microscopy (SEM) can also be used to study silica bodies [40, 41]. The SEM method can be combined with X-ray analysis to provide information on surface structure and composition of silica bodies [42–44]. Here we report a method to study silica bodies using fluorescence microscopy to visualize green autofluorescence in combination with the dry ash-imaging technique. This method was developed using a perennial grass species, *Koeleria macrantha*, commonly known as junegrass, and has potential to be used in all fields of paleobotany and modern plant sciences on silica body research.

## Results and discussion

### Combination of fluorescence microscopy and image software provides an opportunity to study plant silica bodies with high efficiency

**Fig. 1:**
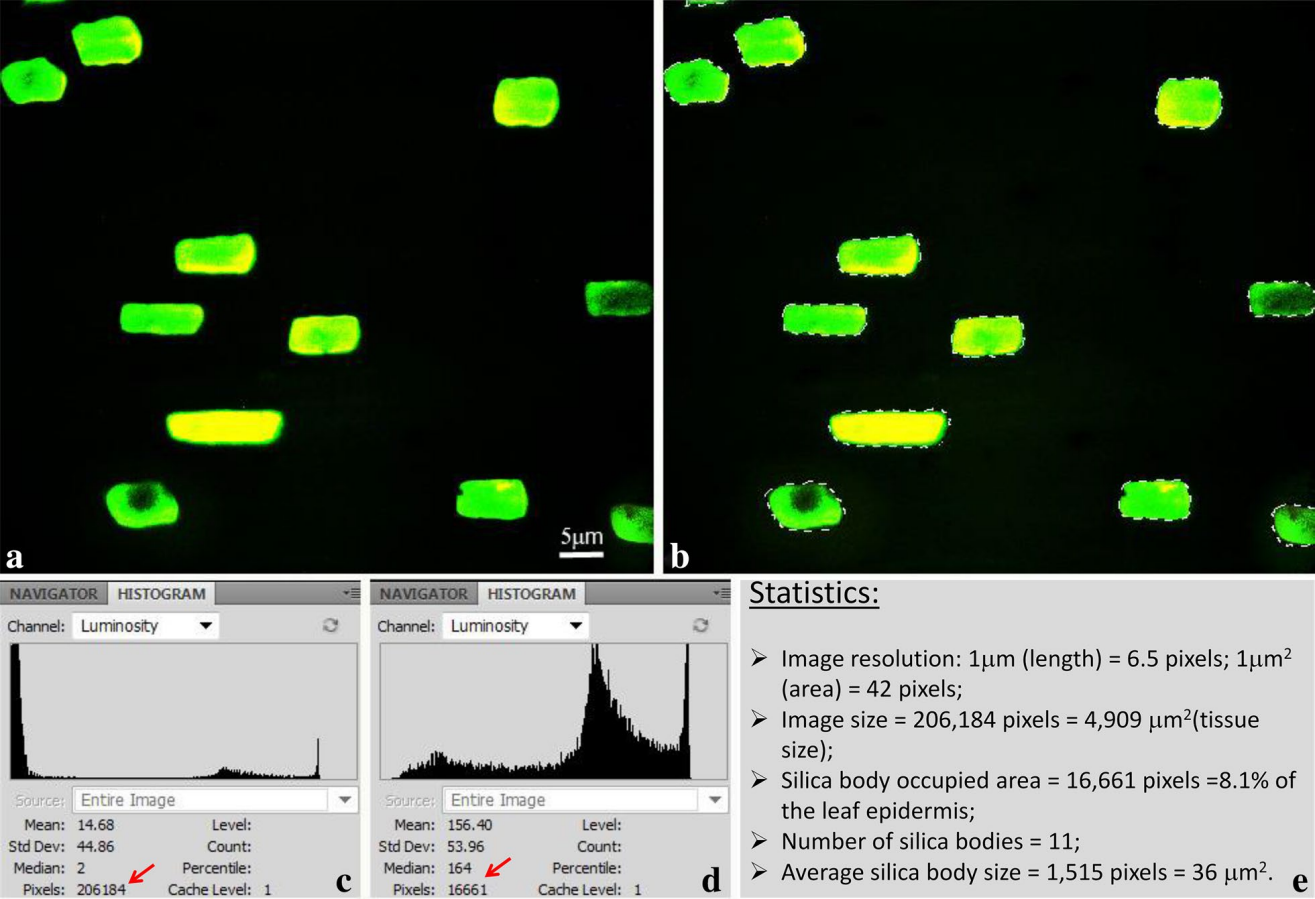
Size and distribution pattern analysis of silica bodies in *K. macrantha* ‘Ireland’ using Adobe Photoshop CS5. **a** A randomly selected microscopy image of ashed leaf sample. **b** The selection of silica bodies using “Magic Wand Tool” (background selection) and “Select->Inverse” tool. **c** Pixel reads of entire image. **d** Pixel reads of the selected silica bodies. **e** A list of statistical results

Silicon-containing structures in plants often display auto- or inducible fluorescence emission that can be examined by fluorescence microscopy [45]. Comparing to brightfield microscopy, the fluorescence emission created ideal conditions for image analysis in high throughput studies, because noisy ash background was eliminated and samples demonstrated clear shape, number, and the distribution pattern of silica bodies (Fig. 1a). Autofluorescence micrographs of an ash-image sample can be easily processed using image software such as Adobe Photoshop and Image J for high-throughput analysis, which include occupancy rate of the leaf epidermis, size and number of silica bodies per unit of leaf surface (Fig. 1a–e). Results can be obtained from a single image or from a random combination of sample images. For example, by analyzing a single image of *K. macrantha* ‘Ireland’, we observed 11 silica bodies in an area of 4909 µm^2^ (Fig. 1), which converted to 2240 silica bodies in an area of 1 square millimeter (2240 sb/mm^2^) abaxial leaf epidermis; the silica bodies occupied 8.1 % of the leaf surface (abaxial). The average size of each silica body was 36 µm^2^ with a standard deviation of 7.94.

**Fig. 2:**
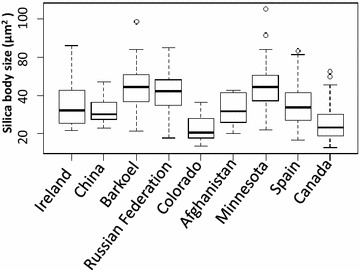
*Box plot* illustrates the size variation of silica bodies in 9 junegrass accessions. *Small circles* demonstrate samples with statistically loud noises

With the same approach we analyzed other accessions using 10 randomly selected images per accession and found that KM-MN and KM-CO exhibited averages of 13,676 sb/mm^2^ and 13,568 sb/mm^2^, respectively, which is approximately 6.1 times more silica bodies per square millimeter comparing to the Ireland accession. The sizes of the silica bodies also differed significantly among accessions; for example, we observed the largest silica bodies with an average size of 52.8 μm^2^ in ‘Barkoel’, whereas the smallest silica bodies with an average size of 26.7 μm^2^ in ‘Canada’ (Fig. 2) [46, 47].

**Fig. 3:**
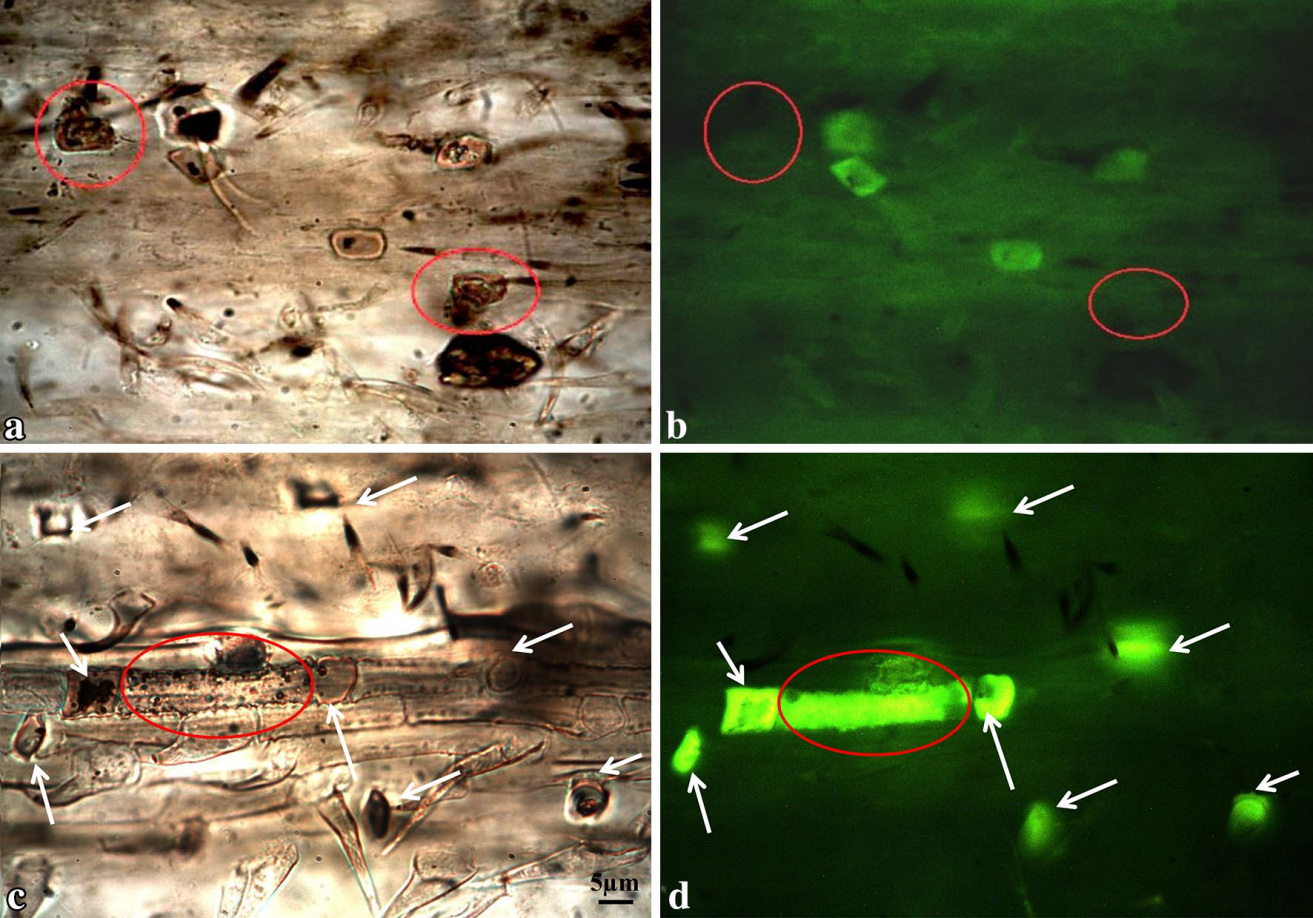
Property differentiation of silica bodies examined by brightfield and fluorescence microscopy. **a** Brightfield and **b** fluorescence microscopic examination of *K. macrantha* ‘Ireland’, showing two types of mineral structures developed in silica cells: Type I structures emitted green autofluorescence; Type II structures did not emit green autofluorescence (*red circled*). **c** Brightfield and **d** fluorescence microscopic examination of *K. macrantha* ‘Russian Federation’, showing that Type I structures developed in silica cells (short cells, *arrows*) emitted autofluorescence. There were also emission of green fluorescence from long cells (*red circle*) defined as type III silica bodies. Images **a**–**d** share the same magnification rate

Three types of silica bodies or silica body related mineral structures were detected by brightfield microscopy (BM) and fluorescence microscopy (FM). Under brightfield microscopy, structures remaining in the ash-imaging process are considered silica bodies [2, 36]. In this study, we compared images from the same ash sample under brightfield and fluorescence microscopy and observed three different types of silica bodies or silica body related structures: Type I silica bodies were developed in the short silica cells and detected under both brightfield and fluorescence microscopy (Fig. 3). Type II silica bodies were also developed in the short silica cells, but only seen under the BM (Fig. 3a), and could not be detected under FM (Fig. 3b). Type III silica bodies were only detected under FM, which were not developed in silica cells (short cells), instead, Type III silica bodies were likely developed in the silicon-enriched long-cells and trichomes (Fig. 3c, d). We frequently observed a number of structures that appeared to look like silica bodies under BM (Fig. 3a), which likely contained less silica and more other chemicals, such as carbon seen in the type I silica bodies as carbon inclusions. However, we were not able to utilize correlative fluorescence microscopy [48] with X-ray analysis in combination with the ash-imaging method to confirm that the intensity of autofluorescence corresponded to the level of silica deposition. Evidence from previous studies using scanning electron microscopy and X-ray analysis suggested that structures developed in silica cells could be classified into two different types based on the silica profile [49, 50]. The majority of silica cells demonstrated significant silica deposition; only a small portion of the short silica cells (~4 %) exhibited silica body-like structures with barely detected silica signal [50].

### Silica bodies presented in both abaxial and adaxial epidermis of *K. macrantha*

**Fig. 4:**
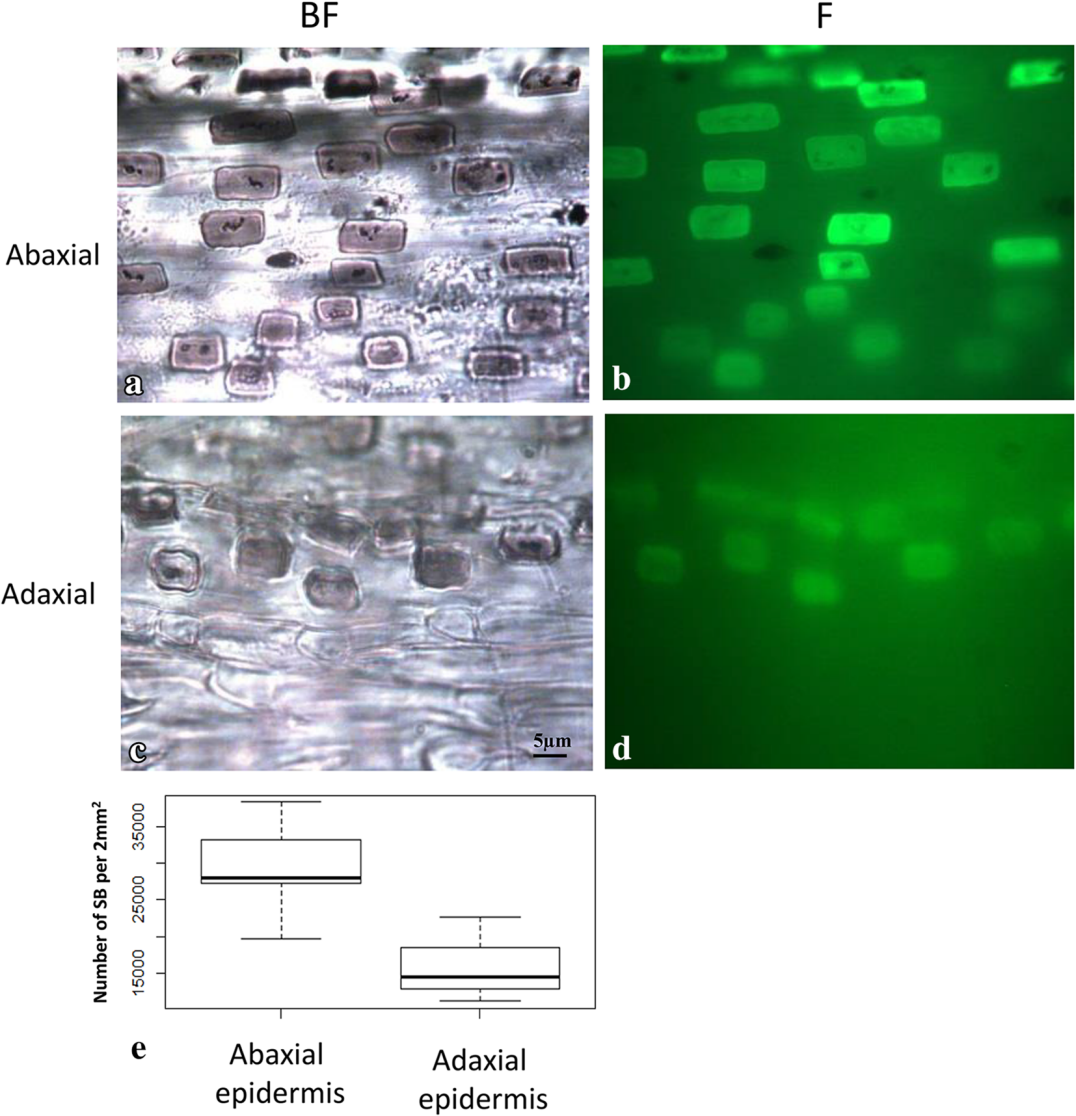
Silica body distribution differences between abaxial and adaxial leaf epidermis of KM-MN. **a** The abaxial epidermis under brightfield microscopy. **b** The abaxial epidermis under fluorescence microscopy. **c** The adaxial epidermis under brightfield microscopy. **d** The adaxial epidermis under fluorescence microscopy. **e** *Box plot* illustrates the distribution differences of silica bodies between abaxial and adaxial leaf epidermis. Images **a**–**d** share the same magnification rate

Most of ash-imaging analysis demonstrated a single layer of silica bodies in grass leaves [2, 36]. By carefully examining the ash-image samples of *K. macrantha*, we found that silica bodies were presented in both abaxial and adaxial leaf epidermis. We observed two layers of silica bodies in some accessions of junegrass at different focus depths of the dry ash slides. Results from the samples that have only one side of epidermal cell layers, either abaxial or adaxial with removal of the opposite epidermis have confirmed that both the abaxial and adaxial epidermis developed silica bodies (Fig. 4). The density, shape, and size of silica bodies in the adaxial epidermis (Fig. 4a), however, differed from those of the abaxial epidermis (Fig. 4b).

**Fig. 5:**
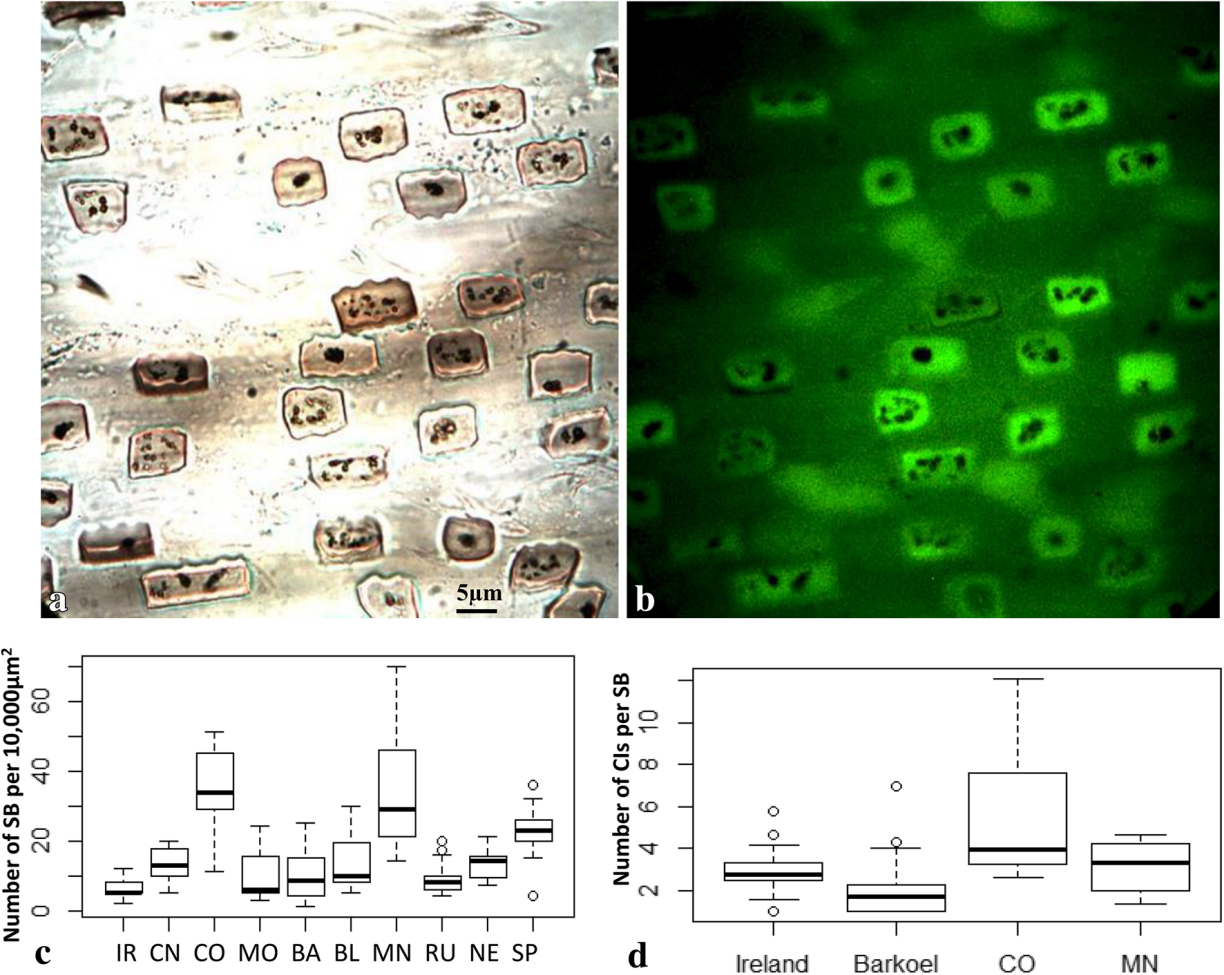
Characterization of carbon inclusions in the silica bodies. **a** Carbon inclusions within silica bodies demonstrated *brown* to *black colored* granules under brightfield microscopy. **b** Carbon inclusions within silica bodies did not emit green fluorescence under fluorescence microscopy. **c** *Box plot* illustrates the distribution differences of silica bodies among 10 accessions (silica body number per 10,000 µm^2^, *white circles* demonstrate loud noises). **d** *Box plot* exhibits the number differences of carbon inclusions per silica body from the selected four accessions (*white circles* indicate statistically loud noises). *IR* ‘Ireland’, *CN* ‘China’, *CO* ‘Colorado’, *MO* ‘Mongolia’, *BA* ‘Barkoel’, *BL* ‘Barleria’, *MN* ‘Minnesota’, *RU* ‘Russian Federation’, *NE* ‘Nebraska’, *SP* ‘Spain’, *CI* carbon inclusion, *SB* silica body. Images **a** and **b** share the same magnification rate

No autofluorescence was detected from carbon inclusions. Carbon inclusions are the residues of organic matter entrapped in silica bodies during the process of silicification [51–53] which can occupy approximately 0.85 % of the total volume of silica bodies [51–53]. Recent studies suggested that the carbon inclusions in plant silica bodies contributed to the enhancement of long-term soil carbon sequestration in agro-ecosystem [54–56]. Nevertheless, we did not observe autofluorescence emitted from the carbon inclusions inside silica bodies (Fig. 5b). The number and distribution pattern of carbon inclusions in silica bodies varied in different *K. macrantha* accessions and cultivars. There was a statistically significant difference in the number of carbon inclusions within silica bodies among accessions: the lowest average was found in ‘Barkoel’ with 2.08 carbon inclusions per silica body and the highest average was found in KM-CO with 5.56 carbon inclusions per silica body (Fig. 5d). Since the number of carbon inclusions in each accession is stable, and the difference between accessions can be used to distinguish the uniqueness in each accession. In addition, the number and size of carbon inclusions can be used to measure the process of cell silicification and the correlation between silica deposition and carbon accumulation [2, 36].

## Conclusions

We propose a method of combining fluorescence microscopy and image processing software for the quantification of silica bodies in *Koeleria macrantha* leaf tissue, which can be applied to biological, ecological and geological studies of grass species. We observed differences between junegrass accessions for both size and density of silica bodies in leaf epidermis. In addition, we identified differences between accessions for carbon inclusions. This study outlines a means to investigate silica bodies in grass models utilizing a novel high throughput method.

## Methods

### Plant material and sample collection

To examine the structure and properties of silica bodies, mature *Koeleria macrantha* leaf blades were collected from plants grown in the greenhouse in a 2:1 mixture of Sunshine MVP (Sungro Horticulture) and MVP:Turface (PROFILE Products LLC) substrates with no additional fertilizer. Plant material was derived from: (a) populations from the University of Minnesota turfgrass breeding program derived from material collected in either Colorado (KM-CO), Nebraska (KM-NE) or Minnesota (KM-MN) [55–57]; (b) the cultivar ‘Barkoel’; (c) several accessions from the United States Department of Agriculture National Plant Germplasm System including PI 430287 (Ireland), PI 387927 (Canada), W6 33040 (Russia Federation), PI 207489 (Afghanistan), W6 13043 (China), and PI 302912 (Spain).

**Fig. 6:**
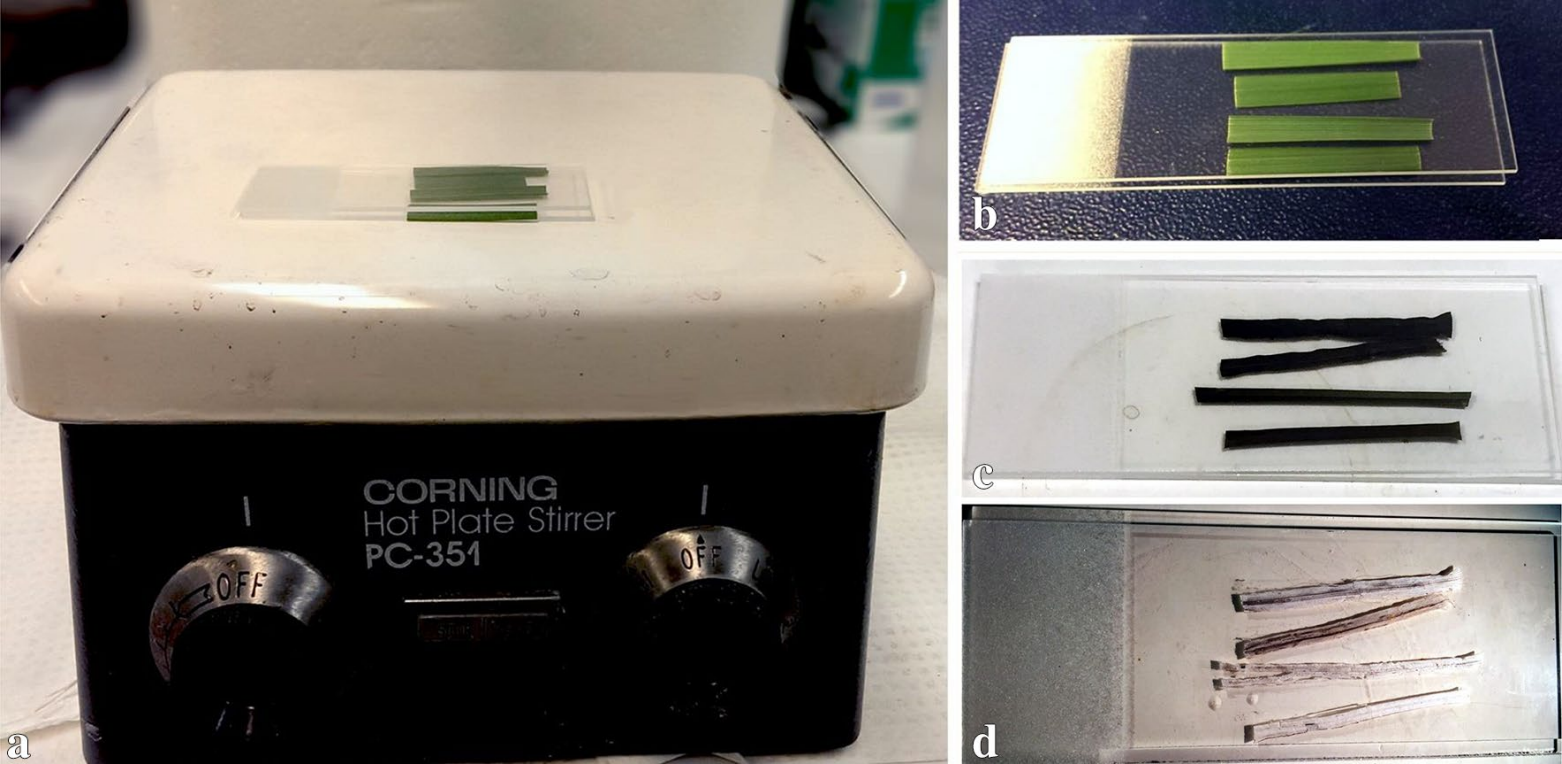
Illustration of sample preparation using dry ash method. **a** Dry ashing was performed by heating leaf-blade samples on a Corning Hot Plate at >608.2 °F/320 °C. **b** Unburned leaf tissues were placed between two microslides. **c** Incompletely burned tissue demonstrated *brown* to *black color*. **d** Completely dry *ashed* tissue showed *gray* to *white color*

The middle section of each leaf blade was cut along the transverse plane and used for ash-imaging sample preparation with removal of the tip and leaf base (Fig. 6b). Each leaf blade was cut into two pieces, one of which was placed on the adaxial and the other on the abaxial side of the leaf onto a glass microslide (VWR Micro Slides were used in this study). On the same microslide, one other leaf from a different plant of the same accession was sampled as a biological replicate (Fig. 6b). A total of 24 plants (thus 12 slides) per accession were analyzed.

### Dry ash-imaging sample preparation

A microslide with the leaf samples on was then covered with another glass microslide in an effort to not disturb the placement of the leaves and to add appropriate weight to keep the ash sample intact (Fig. 6b). The slides were then heated on either a Corning Hot Plate Stirrer PC-351 (Fig. 6a) or a Tek-Pro Heat-Stir 36 H2397-1 (not shown) placed in a fume hood. The hot plate temperature was gradually increased every 5 min up to 320 °C. The temperature was approximated using an infrared thermometer Ryobi IR001 (CW0938) read at >608.2 °F/320 °C. The ash process usually took 2–3 h depending on the accession. Grass leaf samples first turned dark brown or black (Fig. 6c), and then gray (Fig. 6d) when the ashing process completed. To end the heating process, we turned off the hot plate and kept the slides on the plate for 1 h or longer to slowly cool them down. (*Note: The hot plate stirrer and glass slides are extremely hot while preparing the ash samples; do not move the slides directly to a cooler place while they are still hot, which often result in broken slides*.) Slow heating and cooling down prevents the microslides from cracking. For the dry ash samples preparation, we recommend to use a hot plate instead of a coiled electric stove, where the slides often break due to its uneven heating surface.

### Microscopy imaging

After the slides were cooled down for at least 1 h, the top microslide was then carefully removed and discarded. A 1 ml plastic transfer pipette (Fisher Scientific, Pittsburgh, PA, USA) was cut at the 0.5 ml measurement to make an ease cedar wood oil application, and a single drop of cedar wood oil (Electron Microscopy Sciences, Hatfield, PA, USA) was applied. A cover slip was then placed on the microscopy slide without disrupting the sample. Cedar wood oil was allowed to diffuse fully under the cover slip with slightly warming the slide on an alcohol burner. The slides were then imaged using an Ernst Leitz Wetzlar 307143.004 microscope (Wetzlar, Germany) and photographed with a SPOT Insight 4 Camera (Diagnostic Instruments, USA). The auto-fluorescence was detected using a Green Fluorescence Protein filter cube (SN: 31001, excitation at 480 nm, beamsplitter at 505 nm, and emission at 535 nm) that was manufactured by Chroma (Chroma Technology Corp, Bellows Falls, VT, USA). The camera interference program used to take the images was SPOT Basic v4.6. Up to 5 sets of brightfield and fluorescent images per object on the slide were taken at 200× and 800× magnification rate. Duplicate fluorescent images were analyzed using Photoshop CS5 (Adobe Systems Incorporated, San Jose, CA, USA) and/or Image J (imagej.net) for silica body occupancy rate of leaf surface (percentage of surface area), the size of silica bodies, and the pattern of silica body distribution (number of silica bodies per unit) on epidermis. Granule-like structures in silica bodies are carbon inclusions, which were counted and recorded using ImageJ, Analyzing Particles tool. The data collected from 20 silica bodies per accession, each with the number of carbon inclusions and their spatial distribution patterns, such as tightly clustered, loosely clustered, randomly distributed, or single granule.

### Image and data analysis

First, a random selected fluorescence image was imported into Adobe Photoshop CS5 (Fig. 1a). Second, we used the Photoshop Magic Wand Tool to select the dark image background without silica bodies. Third, the Select-Inverse tool was utilized to select all the silica bodies (Fig. 1b). Fourth, we used the Window-Histogram function to read the pixel counts of the entire image (Fig. 1c) and the counts of selected silica bodies (Fig. 1d). To count the number of silica bodies in an image, we used the Window->Tools->Count Tool in the Photoshop CS5, the number of silica bodies was automatically shown. For those who do not have a licensed Adobe Photoshop CS5 or advanced version, the freeware ImageJ is available (http://imagej.nih.gov/ij/download.html) with tutorials videos on YouTube (youtube.com). Using the Analyzing Particles tool in imageJ we could count the number of silica bodies automatically as well. All statistically significant differences were tested at P < 0.05 level. The results were analyzed by ANOVA using R 3.1.2. [58]. The Tukey multiple comparison test was used to test the significant differences of silica bodies among all accessions studied [59].
